# Revisiting the COVID-19 Pandemic: Mortality and Predictors of Death in Adult Patients in the Intensive Care Unit

**DOI:** 10.3390/life14081027

**Published:** 2024-08-19

**Authors:** Adriana Lemos de Sousa Neto, Clesnan Mendes-Rodrigues, Reginaldo dos Santos Pedroso, Denise Von Dolinger de Brito Röder

**Affiliations:** 1 Technical School of Health, Federal University of Uberlândia, Uberlândia 38400902, Brazil; adrianasneto@ufu.br (A.L.d.S.N.); rpedroso@ufu.br (R.d.S.P.); 2 Nursing, Medicine Faculty, Federal University of Uberlândia, Uberlândia 38400902, Brazil; 3 Institute of Biomedical Sciences, Federal University of Uberlândia, Uberlândia 38400902, Brazil; denise.roder@ufu.br

**Keywords:** COVID-19, predictors, mortality, intensive care unit

## Abstract

COVID-19 has generated a global impact due to its contagiousness and high lethality rates, with a large number of deaths occurring in intensive care units (ICUs). This study aimed to verify the occurrence of and understand the factors related to mortality in adult patients with COVID-19 admitted to the ICU in a tertiary hospital. This is a retrospective cohort study, which included COVID-19 patients admitted between March 2020 and December 2021. A total of 588 patients were included, of whom the majority (55.27%) did not survive. Invasive mechanical ventilation was the strongest predictor of the risk of death in the ICU with OR = 97.85 (95% CI = 39.10–244.86; *p* < 0.001), along with age and Simplified Acute Physiology Score 3 (SAPS3). The length of the ICU stay was protective. Evaluating patients on invasive mechanical ventilation in isolation, using an adjusted model, we found the following risk factors: use of vasopressin, renal replacement therapy, red cell distribution width > 15, use of hydrocortisone, and age in years. Protective factors included the days of mechanical ventilation use, being admitted from another service, and being of female sex. Identifying early predictors of mortality in patients with COVID-19 who require hospitalization is essential in the search for actions to prevent and manage complications, which can increase the survival of these patients and reduce the impact on health services.

## 1. Introduction

Coronavirus disease 2019 (COVID-19), declared a pandemic by the World Health Organization (WHO) on 12 March 2020, has generated a global impact due to the high number of deaths, especially among the elderly and those with comorbidities. This has overloaded health systems and culminated in a shortage of intensive care units (ICUs) in hospitals due to the exponential increase in the number of cases [1]. As of 3 March 2024, more than 774 million confirmed cases and more than 7 million deaths have been reported globally [2]. Because it is a highly contagious respiratory disease and involves several factors that affect its severity and mortality, ICU admission is, in many cases, indispensable [3,4].

Clinical and biochemical parameters reflecting acute pulmonary, hepatic, and renal dysfunction, acid-base disturbance, coagulation impairment, and systemic inflammation are among the factors related to the survival of COVID-19 patients in the ICU [5]. Age, gender, hypertension, cardiovascular disease, and chronic kidney disease are also related to the mortality of these patients [4]. The literature points to a mortality rate of around 30% in ICU patients with COVID-19, in addition to the greater susceptibility to infections of this population, which justifies not only establishing conduct aimed at infection control and appropriate antimicrobial management but also seeking clinical evidence of other variables that may contribute to high mortality rates [5,6].

Although the end of the pandemic has already been decreed in May 2023, revisiting the data of specific populations can be essential as a preventive mechanism. Specifically in Brazil, there is a shortage of ICU beds, and the occurrence of healthcare-related infections has increased the length of stay and hospital costs, and reduced the availability of ICU beds for public service systems [7,8]. The population sampled here has shown the occurrence of both co-infections [9] and the early onset of healthcare-associated infections such as catheter-associated bloodstream infections [10]. Patients with COVID-19 have been shown to be more susceptible to infections and secondary complications during hospitalization. Despite this, retrospective studies of the epidemiology of the disease and its associated factors are complex. Few population-based studies are available at the municipal level regarding strains, such as in [11], of the behavior of waves that have clearly been affected by local issues and actions, as in the population sampled in [12], or even the profile and occurrences of the outcomes in these populations; facts that may have made it difficult to manage the pandemic locally.

Reducing and understanding these negative outcomes can strongly affect health systems. Understanding the outcomes of patients who have been infected with the SARS-CoV-2 virus and the high rate of ICU admissions associated with the significant mortality of COVID-19 patients in the first two years of the pandemic will enable future actions in the event of new epidemics of this or other viruses and the development of strategies that can contribute to favorable outcomes. Therefore, this study aimed to verify the occurrence of and understand the predictors of intensive care mortality in adult patients with COVID-19 admitted to the ICU in a tertiary hospital in the inner of Brazil during the pandemic period.

## 2. Methods

### 2.1. Type of Study and Data Collection

This was a retrospective cohort study; the inclusion criteria were patients diagnosed with COVID-19 that was laboratory-confirmed by reverse transcriptase polymerase chain reaction (RT-PCR), aged ≥18 years, and admitted to the ICU of a Brazilian university hospital as a result of the worsening of COVID-19 between March 2020 and December 2021. This teaching hospital has approximately 500 beds and offers highly complex treatment, is tertiary, and is a reference for the macro-region. The research was submitted to and approved by the Human Research Ethics Committee of the University to which the hospital is linked, CAAE: 51805021.5.0000.5152, number 5.043.636/2021.

The following patient characteristics were assessed: age, gender, comorbidities, clinical data such as symptoms on admission to the hospital, duration of symptoms and vital signs on the first day of hospitalization, laboratory results on admission, and treatments carried out in the ICU. All the data were collected electronically from the hospital information system and from the patients’ physical and electronic medical records. The primary outcome assessed here was mortality in the intensive care unit, referred to from now on as mortality.

### 2.2. Data Recoding

For quantitative laboratory tests and blood counts that showed significance and were included in the regression models, the data were also categorized according to the reference values used by the institution. Initially, each exam was classified as below reference value, normal, or as above reference value. In cases where institutional reference values were absent, the cutoff was based on the first or third quartile (e.g., NPR). We performed this analysis to avoid the “bathtub curve” effect in the analyses or differential mortality effects in extremes of distribution. Despite this, in no case was this effect observed. Then, the data were analyzed without categorization and were dichotomized as follows. The laboratory tests were also categorized as normal or abnormal (below or above reference and below the first quartile or above the third quartile) and were tested based on these two levels. However, most of the results were either not different from the absolute values or had a low occurrence in one of the levels (below or above reference). To simplify the analysis and results, not all the tests evaluated are shown here. We maintained for the analyses the absolute value or, in some cases, the dichotomized results, as follows, based on better results. Hematocrit was dichotomized into normal (if ≥35 and ≤45) or non-normal (if <35 or >45). The red blood cell distribution range (RDW) was dichotomized into ≥15 or not (if <15). Prothrombin activation time (PAT) was dichotomized into abnormal (if ≤70) or normal (if >70). The International Normalized Ratio (INR) was dichotomized into abnormal (if ≥1.2) or normal (if <1.2). The neutrophil-to-platelet ratio (NPR) was dichotomized into abnormal (if >58.41) or normal (if ≤58.41).

For the blood count, some ratios or derived indices that have been evaluated in the literature were tested [13,14,15]. The neutrophil/lymphocyte ratio (NLR) was tested by dividing the number of neutrophils by the number of lymphocytes. The platelet/lymphocyte ratio (PLR) was obtained by dividing the number of platelets in thousands (platelets/1000) by the number of lymphocytes. The derived neutrophil/lymphocyte ratio (d-NLR) was obtained using the equation d-NLR = N/([L × 1000] − N), where N is the number of neutrophils and L is the number of lymphocytes. The monocyte/lymphocyte ratio (MLR) was calculated by dividing the number of monocytes by the number of lymphocytes. The neutrophil/platelet ratio (NPR) was the number of neutrophils divided by the number of platelets. The systemic immuno-inflammation index (SII) was obtained using the equation SII = (N × P)/(L × 1000), where N is the number of neutrophils, P is the number of platelets, and L is the number of lymphocytes. In both cases, multiplying by 1000 served to improve the scale of the variable [13,14,15].

### 2.3. Statistical Analysis

The analyses and tests were carried out separately for patients on invasive mechanical ventilation (IMV) and for all patients together (see results). One of the most important points justifying this approach is the presence of few deaths in the group that did not receive IMV (five deaths out of 164 patients), which would not allow us to adequately estimate the statistical trend values and the associations to be tested. In the group that received IMV, there were 320 deaths out of 424 patients. IMV alone is already the main risk factor for mortality in COVID-19 patients (see results).

For the quantitative data, the median, first quartile, and third quartile were calculated, given the lack of normality assessed by the Kolmogorov–Smirnov Lilliefors test. For the qualitative data, the relative frequency in percentage and its 95% confidence interval (95% CI) were calculated for each level of the variables. To compare the ICU death and survivor groups in the association analyses, the Likelihood ratio test was used for qualitative variables and the Mann–Whitney test for quantitative variables. We used the likelihood ratio test for contingency tables as proposed by [16]. Simple and multiple logistic regression analyses were used to predict the occurrence of death in the ICU. For the logistic regression models, the obesity variable was chosen due to the lower sample loss in relation to weight. When necessary, the variables were dichotomized for better estimation. Simple regression models (unadjusted) were built for all available variables in both cases. Due to insufficient data in the records, multiple models could not be built for all variables. For the multiple models, priority variables were chosen with sufficient sample sizes, the presence of representative deaths and survivors, and without dependence on others. In addition, for the (adjusted) multiple regression, models were built only for the variables that had already shown a significant difference in the previous association analyses and were not dependent on others. The multiple models were presented in full and reduced form. The Wald test probability (*p*-value < 0.05) and the backward method were used to select the variables in the model. In addition, the odds ratio and its 95% confidence interval were calculated for all models, unadjusted (or crude) or adjusted. All the analyses were carried out using SPSS software version 20.0. A 5% significance level was adopted for all analyses.

## 3. Results

The study included 588 adult patients admitted to the ICU, of whom 55.27% died (95% CI = 51.25–59.29; 325/588). Among all the patients, 164 did not use IMV and 424 did. Among the patients who did not use IMV, 3.05% died (95% CI = 0.42–5.68; 5/164). Among the patients who used IMV, 75.47% died (95% CI = 71.38–79.57; 320/424). Given these differences in mortality associated with IMV, the patients were allocated into two groups for statistical analysis. The first group was the general group, which included all patients, and the second group included only patients who received IMV. In addition, exploratory analyses demonstrated the dependence between some variables, such as patients who received IMV were also those who used vasoactive drugs, used sedatives, received invasive procedures, and used medications or underwent interventions more commonly associated with mortality and/or worsening of the condition.

In the general group, which included all patients, female gender proved to be a protective factor OR = 0.62 (95% CI = 0.44–0.87; *p* = 0.005). The median age was 53 years (IQR = 40.5–65.5) for survivors and 65 (IQR = 52–73) for non-survivors (*p* < 0.001). The occurrence of diabetes mellitus was higher in the group that died (32.92 versus 19.01%), proving to be a risk factor OR = 2.09 (95% CI = 1.42–3.07, *p* < 0.001). ICU stay in days had a median of 8 (IQR = 4–17) for survivors and 11 (IQR = 6–22) for non-survivors (*p* < 0.001), which was similar behavior to the length of the hospital stay with a median of 19 and 15 days, respectively (*p* < 0.001). Survivors had a median Simplified Acute Physiology Score 3 (SAPS 3)—a score that assesses the severity of the patient in the first hour of admission—of 49 (IQR = 38–58) and non-survivors 61 (IQR = 49–71) (*p* < 0.001). Patients who died also had more comorbidities than survivors, despite the same median (Table 1).

Protective factors for patients who received IMV were being admitted from another service OR = 0.59 (95% CI = 0.36–0.97; *p* = 0.033) and receiving a tracheotomy OR = 0.24 (95% CI = 0.14–0.39; *p* < 0.001), although in the latter case, the association was due to the longer duration of IMV use in the surviving patients. In the group receiving IMV, female gender was a protective factor OR = 0.36 (95% CI = 0.23–0.56; *p* < 0.001). The median age was 49.50 years (IQR = 38–61) for survivors and 64 (IQR = 51–72) for non-survivors (*p* < 0.001). Diabetes mellitus almost doubled the risk of death (OR = 1.96 [95% CI = 1.15–3.33; *p* = 0.010]). Smoking acted as a predictor of mortality in the unadjusted model (OR = 2.35 [95% CI = 1.22–4.52; *p* = 0.006]), as did the use of noradrenaline, vasopressin, and hydrocortisone; all three variables had a *p*-value < 0.001. With an increase of more than six times in the risk of death, OR = 6.29 (95% CI = 3.65–10.85; *p* < 0.001), renal replacement therapy was also among the predictors of mortality in the unadjusted models (Table 2). ICU stay in days had a median of 21.5 (IQR = 13–33.5) for survivors and 12 (IQR = 6–22) for non-survivors (*p* < 0.001). Survivors had a median SAPS 3 score of 51 (IQR = 37.75–62) and non-survivors 61 (IQR = 49–71) (*p* < 0.001). The duration of IMV use in days was a protective factor, as there was greater survival among patients who used IMV for longer (Table 3). This may be related to the greater severity of the disease in some patients at the time of admission to the ICU, who presented with severe respiratory failure and died shortly after being put on IMV. Those patients who managed to overcome the first few days of greater severity of the lung condition were able to benefit from intensive treatment with the help of IMV and survive the disease.

IMV was the strongest predictor of the risk of death in the ICU in the unadjusted models with OR = 97.85 (95% CI = 39.10–244.86; *p* < 0.001). Based on this, a prediction model was proposed for all patients (Table 4) and another only for patients on IMV (Table 4). The use of mechanical ventilation was also associated with greater use of vasoactive drugs and use of devices; as there were few deaths in the group without IMV, it was not possible to build models that took this interaction into account (use or not of IMV and death in the ICU).

After applying the multiple logistic regression model, the overall group had as predictors of mortality the use of IMV, OR = 306.74 (95% CI = 87.47–1075.71; *p* < 0.001), age, OR = 1.04 (95% CI = 1.03–1.06; *p* < 0.001), and SAPS 3 score, OR = 1.03 (95% CI = 1.01–1.04; *p* = 0.001). The length of stay in the ICU in days was a protective factor in this group OR = 0.96 (95% CI = 0.85–0.98; *p* < 0.001) (Table 4).

When the adjusted model was applied to the group receiving IMV, risk factors were age, OR = 1.03 (95% CI = 1.01–1.05; *p* = 0.006), use of vasopressin, OR = 7.87 (95% CI = 3.54–17.46; *p* < 0.001), use of hydrocortisone, OR = 2.33 (95% CI = 1.05–5.16; *p* = 0.038), RDW > 15, OR = 3.84 (95% CI = 1.60–9.21; *p* = 0.003), and renal replacement therapy OR = 5.42 (95% CI = 2.55–11.51; *p* < 0.001) as predictors of mortality. In this group, the protective factor was days of use of mechanical ventilation, OR = 0.95 (95% CI = 0.93–0.97; *p* < 0.001), being admitted from another service, OR = 0.43 (95% CI = 0.21–0.86; *p* < 0.001), and being female, OR = 0.42 (95% CI = 0.22–0.82; *p* < 0.001) (Table 4).

## 4. Discussion

In this study, the majority (55.27%) of COVID-19 patients admitted to the ICU did not survive. A review that evaluated different studies published during the pandemic found that the mortality percentage for COVID-19 patients in the ICU reached 84.6%. This high percentage may have been due to the severity of the disease, the population served, comorbidities, difficulties faced by health systems, and socioeconomic status [17,18]. As the pandemic progressed, mortality prevalences fell to close to 40%, a fact that may be related to the rapid availability of scientific studies related to guidelines for the clinical management of COVID-19 [17] and the advent of vaccines in record time, allied with their effectiveness [19].

The SAPS3 score was effective in predicting mortality in the general group of patients. This score shows good applicability in predicting mortality in ICUs and in patients with COVID-19, in patients admitted to both private Brazilian ICUs [20] and public institutions [21]. The score should be used with caution, and future studies should test its calibration for each population studied and for specific diseases, taking into account other comorbidities, such as diabetes, since they can interfere with the calibration result [22].

Age was associated with higher mortality as an independent factor, both in the group of patients who received IMV and in the general group, which corroborated data from a meta-analysis of COVID-19 cases from five countries, which concluded that age was an important predictor of mortality in this population [23]. In Brazil, the age factor was also preponderant in mortality indices; although aspects of the municipality were also essential in defining mortality [24], probably related to differences in the local management of the pandemic or the conditions of the municipal health network. Another important factor was the higher mortality in men who received IMV. There are indications of higher mortality in men compared to women, which is even more prevalent in older people, although the causal effects are still unknown and are probably related to the virus infection itself [25].

Some comorbidities seem to play an important role in the clinical evolution and outcomes of COVID-19. Diabetes mellitus acted as a predictor of mortality in the univariate analysis of this study, both for the general group and for the group of patients who received IMV. The high mortality from COVID-19 in patients with diabetes can be explained by some processes, such as the permissibility of pluripotent stem cells derived from pancreatic beta cells for infection by SARS-CoV-2 [26]. There is also greater insulin resistance in COVID-19 patients due to the exaggerated action of angiotensin II, and insulin resistance triggers the activation of the inflammatory response and the cytokine storm [27]. Thus, the release of different inflammatory mediators into the blood caused by SARS-CoV-2 is exacerbated in patients with diabetes [13,28]. The analysis of pancreatic autopsies of patients infected with COVID-19 showed that beta cells were infiltrated by SARS-CoV-2 in all patients [29]. COVID-19 patients with better glycemic control have better outcomes [30]. Diabetic patients are also those with the highest risk of using mechanical ventilation (adjusted OR = 2.20; *p* = 0.004; 95% CI: 1.29–3.75), and this may justify not including them in the adjusted models, since the impact of IMV overlaps with other variables. Other comorbidities may also be important, as we observed that an increase in the number of comorbidities increases the risk of death, as seen in the literature. However, due to the low representativeness and experimental difficulties, assessing the impact of each of them may be complex [31]. Further studies, such as case-control studies, should assess the increased risk of death from the comorbidities that we found to play a significant independent role here, such as cardiovascular disease presence, chronic obstructive pulmonary disease presence, chronic kidney disease presence, smoking habit presence, and correcting for confounding variables for each comorbidity.

The use of IMV was the strongest independent predictor of mortality in the patients evaluated (OR = 97.85) and should receive more attention in the clinical management of the patient. Mechanical ventilation is necessary as a support measure for many COVID-19 patients with acute respiratory syndrome and directly reflects the severity of the disease. The use of non-invasive mechanical ventilation did not prove to be a protective factor in our study, possibly due to the rapid and progressive evolution of the pulmonary condition in these patients. Despite the possible complications inherent with IMV, such as barotrauma with alveolar rupture and superimposed bacterial pneumonia, this therapy was a widely used strategy in the clinical management of patients. These complications can be minimized if imaging tests are carried out frequently, in addition to rigorous monitoring of the patient’s clinical condition, with a view to early diagnosis of pneumonia and complications that would lead to appropriate management [32]. In this sense, the presence of other comorbidities with significant effects observed by us, such as chronic obstructive pulmonary disease presence and smoking habit presence, could reinforce this lethality. On the other hand, it is important to identify the ideal time to introduce mechanical ventilation, since its late installation can reduce patient survival. A multicenter study that evaluated about 1900 patients with COVID-19 in the ICU in the United States concluded that early initiation of mechanical ventilation reduces the chance of mortality compared to those patients who receive late intervention [33]. Patients died with a median of 11.5 days of mechanical ventilation use, and survivors had a median of 15.5 days under IMV. The prospective cohort observed that survivors had a median of 27 days of IMV use and non-survivors had a median of 10 days of use [34].

In the analysis of patients in the general group, the length of stay in the ICU acted as a protective factor against mortality; that is, the longer the stay in the ICU, the lower the chance of mortality. The justification for this finding may be due to the extreme severity of the clinical picture of COVID-19 for some patients admitted to the ICU, leading to early death, mainly in the first two years of the pandemic. Another factor to discuss is that the duration in the ICU or of hospitalization in these cases can also work as an associated outcome and not a predictor. Those patients who survived the first days of hospitalization received the appropriate intensive care necessary for health recovery. However, a long time in the ICU increases the risk of other unfavorable outcomes, such as the risk of healthcare-associated infections and impairing the mental health and family relationships of patients, in addition to the higher cost and burden on health systems [35,36], which consequently increase the risk of death. In Brazil, the occurrence of healthcare-associated infections has already been clearly related to overload and increased health costs for the public health system [7,8]. Thus, the length of stay in the ICU is an important measure for planning the capacity of beds and hospital resources if SARS-CoV-2 presents a seasonal pattern [37]. Reducing hospitalization time and preventing healthcare-associated infections are important indicators for health services. In the patients evaluated here, we observed both the presence of infections secondary to COVID-19, such as aspergillosis [9], and the early installation of healthcare-associated infections, such as catheter-associated bloodstream infection [10]. It is difficult to define the presence and classification of healthcare-associated infections based on international criteria in Brazil since compulsory notifications follow the criteria of Brazilian regulatory bodies. Despite this, we observed positive cultures for microorganisms in blood, urine, and tracheal aspirate).

Patients with COVID-19 who have acute kidney injury have a substantially increased risk of death, especially if they require renal replacement therapy [38], an association that was observed in this study in patients admitted with high levels of creatinine and also in the group of patients on IMV who received renal replacement therapy, according to the multiple logistic regression analysis. Vasopressin is commonly used in respiratory failure caused by COVID-19 to replace other drugs because it does not impair renal function and does not cause overload in the right ventricle [39]. However, in patients with non-septic shock, vasopressin associated with catecholamines should be avoided because it is related to possible hyponatremia and volume overload [40]. In the group of IMV patients in this study, vasopressin increased the risk of death by almost eight times (OR = 7.87).

The use of hydrocortisone was associated with higher mortality in the group of patients on IMV (OR = 2.33). However, a meta-analysis that included seven randomized controlled trials with a total of 1703 patients concluded that mortality was lower among patients receiving corticosteroids, including dexamethasone, hydrocortisone, or methylprednisolone, compared to those who received placebo or conventional treatment [41]. The infusion of intravenous hydrocortisone in patients with severe COVID-19 reduces the amount of interleukin 6 in the lungs, thus reducing the inflammation that occurs in the acute respiratory syndrome related to COVID-19 [42]. The findings of this study may be related to the late commencement of hydrocortisone therapy. However, this hypothesis was not analyzed due to the lack of accurate data that confirmed the exact date on which the drug was introduced for the treatment of patients. Retrospective studies of the impact of using one drug or another are still complex in Brazil, given the low quality of patient records in the medical record. Digital and structured data in electronic health records in Brazil are incipient [43].

In the analysis of blood counts, the group of patients who received IMV had RDW > 15 as a predictor of mortality (OR = 3.84). The dysfunction in the size and aggregability of erythrocytes contributes to the impairment of capillary blood flow and microangiopathy/microthrombosis in patients with COVID-19 [44]. RDW, platelets, and leukocytes are biomarkers that can predict disease progression and mortality in patients with COVID-19. Thus, these combined parameters deserve special attention from the beginning of hospitalization in future waves of COVID-19 [45]. Altered markers for platelets, abnormal neutrophil to platelet ratio, abnormal prothrombin activation time, and abnormal International Normalized Ratio were independently associated with a higher risk of mortality and reflect changes in the coagulation pattern of patients as observed in the literature [46,47]. This is probably associated with vascular endothelial cell dysfunction—a hyper-inflammatory immune response—and hypercoagulability [48]. Markers related to hemograms and their indices or derived reasons have had diverse findings in the literature but have shown great potential for predicting outcomes [13,14,15]. In Brazil, an NLR greater than 10 has been associated with higher mortality, either independently or associated with D-dimer [49], although there is no consensus on the reference values for most of these indices.

At the height of the COVID-19 pandemic and with the increasing number of cases and deaths from the disease, researchers were looking for biomarkers upon patient admission that could predict mortality. In this study, in the group receiving IMV, high levels of lactic dehydrogenase (LDH), D-dimer, and creatinine (*p* = 0.016, *p* = 0.009, *p* < 0.001, respectively) were associated with mortality. However, when multivariate logistic regression models were applied, these variables did not have statistical significance alone. These patients with high values for the tests are probably also those who received IMV and/or had other risk factors for death. Elevated levels of LDH on admission are associated with an approximately six-fold increase in the development of severe disease and a 16-fold increase in the chances of mortality in patients with COVID-19 [50]. Serum LDH is a prognostic marker of pulmonary injury, constituting a rapid, effective, and accessible method for predicting a higher risk of mortality in patients with COVID-19, and its measurement should be prioritized to enhance actions aimed at reducing mortality in these patients [51].

The level of serum D dimer collected on admission is a product of fibrin degradation and reflects the activation of coagulation and fibrinolysis, being a biomarker used to predict the mortality of patients with COVID-19 [52]. A meta-analysis that evaluated 2911 patients concluded that increased rates of this biomarker can increase the risk of death by up to four times [53]. Serum creatinine level at admission can predict mortality in patients with COVID-19, and the literature points to 1.12 mg/dL as a cutoff point for this prediction [54]. Our study found a median of 1.22 mg/dL of creatinine among the non-survivors who received IMV when an unadjusted statistical model was applied. The literature points out a nearly fourfold higher incidence of mortality in patients with values of procalcitonin > 0.28 ng/mL during their hospital stay [55]. Unfortunately, however, we found that these data were added to the medical records of only a few patients in the sample, which made the statistical analysis of this biomarker as a predictor of mortality unfeasible.

Some aspects that could affect mortality rates in the population studied here are difficult to control. There are records of the differential distribution of the incidence of COVID-19 in different neighborhoods of the city [56], which could reflect socio-economic differences and access to health services. Studies of the behavior of the waves and the prevalence of the strains in the municipality are absent or do not cover the entire pandemic period, and the municipality did not follow the guidelines for the virus contention recommended by the state, which clearly affected the occurrence of cases in the municipality [11,12]. Thus, these confounding factors could not be corrected in our study. The hospital is also a macroregional reference, with patients from different municipalities—information that is not always present in the medical records. Compulsory notification data from the municipalities included in the microregion of Uberlandia in the initial phase of the pandemic had already demonstrated the impact of the size of the municipality of origin (small, medium, or large) and decentralized policies on controlling the spread of the pandemic [57].

This study has important limitations because it is single-center, presents great data loss of some variables not contained in medical records, and does not include a prospective follow-up with a control group without COVID-19, which prevented in-depth analysis and generalization of results. The results found, however, contribute to the elucidation of factors associated with intensive care unit mortality, directing future research to control the possible confounders and providing the creation of intensive care protocols for patients with COVID-19 to mitigate mortality in this population. Local data are of paramount importance for the management of the municipalities since they may present marked differences in behavior in relation to the state or even the country.

## 5. Conclusions

Most patients with COVID-19 admitted to the ICU did not survive. IMV was a risk predictor strongly associated with mortality along with age, diabetes mellitus, and high SAPS 3 score for the general group of patients. In the group of patients receiving IMV, age, vasopressin, RDW > 15, renal replacement therapy, and hydrocortisone use were associated with mortality. Early identification of predictors of mortality in patients with COVID-19 that require hospitalization in the ICU is paramount in the search for strategies to prevent and manage complications, which may increase the survival of these patients and reduce the pressure on health services.

## Figures and Tables

**Table 1:** Some admission variables related to all survivors and deaths in patients with COVID-19 admitted to an adult intensive care unit.

	% Yes (95% Confidence Interval) [n]		*p*-Value	Unadjusted Odds-Ratio (95% Confidence Interval)
Trait	Survivor (n = 263)	Non-Survivor (n = 325)		
Admitted from another service	67.30 (61.63–72.97) [177]	63.38 (58.15–68.62) [206]	0.321	0.84 (0.60–1.19)
Female sex	45.25 (39.23–51.26) [119]	33.85 (28.70–38.99) [110]	0.005	0.62 (0.44–0.87)
Obesity presence	35.74 (29.95–41.53) [94]	32.62 (27.52–37.71) [106]	0.427	0.87 (0.62–1.23)
Systemic arterial hypertension presence	48.29 (42.25–54.33) [127]	53.23 (47.81–58.66) [173]	0.233	1.22 (0.88–1.69)
Diabetes mellitus presence	19.01 (14.27–23.75) [50]	32.92 (27.81–38.03) [107]	<0.001	2.09 (1.42–3.07)
Cardiovascular disease presence	10.65 (6.92–14.37) [28]	12.92 (9.28–16.57) [42]	0.395	1.25 (0.75–2.07)
Asthma presence	1.90 (0.25–3.55) [5]	1.54 (0.20–2.88) [5]	0.736	0.81 (0.23–2.82)
Chronic obstructive pulmonary disease presence	7.60 (4.40–10.81) [20]	11.38 (7.93–14.84) [37]	0.120	1.56 (0.88–2.76)
Chronic kidney disease presence	7.22 (4.10–10.35) [19]	10.15 (6.87–13.44) [33]	0.210	1.45 (0.81–2.62)
Etilism habit presence	4.94 (2.32–7.56) [13]	8.00 (5.05–10.95) [26]	0.134	1.67 (0.84–3.32)
Smoking habit presence	18.63 (13.93–23.34) [49]	23.08 (18.5–27.66) [75]	0.187	1.31 (0.88–1.96)
COVID-19 vaccine previous hospital admission	17.11 (12.56–21.66) [45]	20.31 (15.93–24.68) [66]	0.323	1.23 (0.81–1.88)
Invasive mechanical ventilation use	39.54 (33.63–45.45) [104]	98.46 (97.12–99.80) [320]	<0.001	97.85 (39.1–244.86)
	Median (Quartile 1–Quartile 2) [n]		*p*-value	Odds-Ratio (95% Confidence interval)
Trait	Survivor (n = 263)	Non-survivor (n = 325)		
Age in years	53 (40.5–65.5) [263]	65 (52–73) [325]	<0.001	1.03 (1.02–1.04)
Total number of comorbidities	1 (0–2) [263]	1 (1–2) [325]	0.007	1.19 (1.03–1.36)
Time in days from symptom to ICU admission	11 (8–14) [245]	11 (7–14) [285]	0.229	0.99 (0.96–1.02)
Simplified Acute Physiology Score 3 score	49 (38–58) [263]	61 (49–71) [325]	<0.001	1.05 (1.04–1.06)
Simplified Acute Physiology Score in %	15.9 (6–31.5) [263]	39.8 (19–58.5) [325]	<0.001	1.04 (1.03–1.05)
Length of stay at the ICU in days	8 (4–17) [263]	11 (6–22) [325]	<0.001	1.02 (1.00–1.03)
Length of stay at the Hospital in days	19 (11–31) [263]	15 (7–27) [325]	<0.001	

**Table 2:** Some categorical variables related to survivors and deaths in patients in invasive mechanical ventilation with COVID-19 admitted to an adult intensive care unit.

	% Yes (95% Confidence Interval) [n]		*p*-Value	Unadjusted Odds-Ratio (95% Confidence Interval)
Trait	Survivor	Non-Survivor		
Admitted from another service	74.04 (65.61–82.46) [77]	62.81 (57.52–68.11) [201]	0.033	0.59 (0.36–0.97)
Female sex	57.69 (48.2–67.19) [60]	32.81 (27.67–37.96) [105]	<0.001	0.36 (0.23–0.56)
Obesity presence	38.46 (29.11–47.81) [40]	33.13 (27.97–38.28) [106]	0.322	0.79 (0.5–1.25)
Systemic arterial hypertension presence	45.19 (35.63–54.76) [47]	53.13 (47.66–58.59) [170]	0.160	1.37 (0.88–2.14)
Diabetes mellitus presence	20.19 (12.48–27.91) [21]	33.13 (27.97–38.28) [106]	0.010	1.96 (1.15–3.33)
Cardiovascular disease presence	7.69 (2.57–12.81) [8]	12.81 (9.15–16.47) [41]	0.140	1.76 (0.8–3.89)
Asthma presence	0 (0–0) [0]	1.56 (0.2–2.92) [5]	0.092	
Chronic obstructive pulmonary disease presence	4.81 (0.70–8.92) [5]	11.56 (8.06–15.07) [37]	0.032	2.59 (0.99–6.77)
Chronic kidney disease presence	2.88 (0–6.1) [3]	10.31 (6.98–13.64) [33]	0.009	3.87 (1.16–12.9)
Etilism habit presence	5.77 (1.29–10.25) [6]	8.13 (5.13–11.12) [26]	0.417	1.44 (0.58–3.61)
Smoking habit presence	11.54 (5.40–17.68) [12]	23.44 (18.8–28.08) [75]	0.006	2.35 (1.22–4.52)
COVID-19 vaccine previous admission	13.46 (6.90–20.02) [14]	20.31 (15.9–24.72) [65]	0.109	1.64 (0.88–3.06)
Blood transfusion	26.92 (18.40–35.45) [28]	31.56 (26.47–36.65) [101]	0.368	1.25 (0.76–2.05)
Use of noradrenaline	90.38 (84.72–96.05) [94]	99.38 (98.51–100.00) [318]	<0.001	16.92 (3.64–78.55)
Use of vasopressin	17.31 (10.04–24.58) [18]	70.94 (65.96–75.91) [227]	<0.001	11.66 (6.65–20.47)
Use of hydrocortisone	29.81 (21.02–38.6) [31]	71.56 (66.62–76.51) [229]	<0.001	5.93 (3.65–9.63)
Use of neuroblocker	71.15 (62.45–79.86) [74]	68.13 (63.02–73.23) [218]	0.560	0.87 (0.53–1.41)
Use of midazolam	94.23 (89.75–98.71) [98]	91.25 (88.15–94.35) [292]	0.315	0.64 (0.26–1.59)
Use of fentanyl	98.08 (95.44–100.72) [102]	93.44 (90.72–96.15) [299]	0.045	0.28 (0.06–1.21)
Use of propofol	59.62 (50.19–69.05) [62]	51.25 (45.77–56.73) [164]	0.136	0.71 (0.46–1.12)
Use of ketamine	37.5 (28.20–46.8) [39]	44.06 (38.62–49.5) [141]	0.238	1.31 (0.83–2.07)
Use of non-invasive ventilation	62.5 (53.20–71.8) [65]	61.56 (56.23–66.89) [197]	0.864	0.96 (0.61–1.52)
Use of indwelling bladder catheter	100 (100–100) [104]	97.19 (95.38–99.00) [311]	0.024	
Use of tracheostomy	40.38 (30.95–49.81) [42]	13.75 (9.98–17.52) [44]	<0.001	0.24 (0.14–0.39)
Use of central venous catheter	100 (100–100) [104]	98.75 (97.53–99.97) [316]	0.132	
Renal replacement therapy	18.27 (10.84–25.7) [19]	58.44 (53.04–63.84) [187]	<0.001	6.29 (3.65–10.85)
Haematocrit abnormal	33.65 (24.57–42.74) [35]	48.28 (42.79–53.76) [154]	0.009	1.84 (1.16–2.82)
Red cell distribution width >15	13.46 (6.9–20.02] [14]	27.59 (22.68–32.49] [88]	0.002	2.45 (1.32–4.53)
Neutrophil to platelet ratio abnormal	17.48 (10.14–24.81] [18]	33.54 (28.34–38.75] [106]	0.001	2.38 (1.36–4.17)
Prototombin activation time abnormal	6.12 (1.38–10.87] [6]	19.02 (14.61–23.42] [58]	0.001	3.60 (1.50–8.63)
International Normalized Ratio abnormal	5.1 (0.75–9.46] [5]	15.84 (11.73–19.95] [48]	0.003	3.50 (1.35–9.06)

**Table 3:** Some quantitative variables related to survivors and deaths in patients in invasive mechanical ventilation with COVID-19 admitted to an adult intensive care unit.

	Median (Quartile 1–Quartile 2) [n]		*p*-Value	Unadjusted Odds-Ratio (95% Confidence Interval)
Trait	Survivor	Non-Survivor		
Age in years	49.50 (38–61) [104]	64.00 (51–72) [320]	<0.001	1.05 (1.03–1.06)
Total number of comorbidities	1 (0–2) [104]	1 (1–2) [320]	0.003	1.31 (1.08–1.6)
Time in days from symptom to ICU admission	11 (8–13.75) [98]	11 (7–14) [282]	0.676	0.99 (0.95–1.03)
Length of stay at the ICU in days	21.5 (13–33.5) [104]	12 (6–22) [320]	<0.001	0.97 (0.96–0.98)
Simplified Acute Physiology Score 3 score	51 (37.75–62) [104]	61 (49–71) [320]	<0.001	1.04 (1.02–1.05)
Simplified Acute Physiology Score in %	20.25 (6–39.8) [104]	39.8 (19–58.5) [320]	<0.001	1.03 (1.02–1.04)
Days of mechanical ventilation use	15.5 (9–27.25) [104]	11.5 (5–19) [312]	<0.001	0.98 (0.97–1.00)
Hemoglobin in g/dL	12.6 (11.18–14.13) [104]	12.4 (10.8–14.05) [319]	0.641	0.97 (0.88; 1.06)
Leukocytes in 1000/mm^3^	11.3 (7.58–13.7) [104]	11.9 (8.4–17.05) [319]	0.056	1.05 (1.01–1.09)
Haematocrit in %	37.65 (34.18–41.58) [104]	37.3 (32.7–41.55) [319]	0.631	0.99 (0.96–1.02)
Mean Corpuscular Volume in fL	88.9 (85.35–91.2) [104]	88.9 (85.1–93.2) [319]	0.275	1.02 (0.99–1.05)
Mean Corpuscular Hemoglobin in pg	29.65 (28.8–30.6) [104]	29.9 (28.6–31.1) [319]	0.219	1.06 (0.97–1.16)
Mean Corpuscular Hemoglobin Concentration in g/dL	33.5 (32.2–34.63) [104]	33.6 (32.45–34.6) [319]	0.873	0.98 (0.86–1.13)
Red cell distribution width in %	13.9 (13.2–14.6) [104]	14.1 (13.2–15.2) [319]	0.097	1.16 (1.00–1.34)
Mean platelet volume in fL	10.5 (10–11.1) [103]	10.7 (10–11.4) [314]	0.405	1.05 (0.84–1.32)
Myelocytes in units by mm^3^	0 (0–0) [104]	0 (0–0) [319]	0.590	1.00 (1.00–1.00)
Rods in units by mm^3^	601 (298–1349) [104]	755 (377.5–1444.5) [319]	0.177	1.00 (1.00–1.00)
Segmented in units by mm^3^	8406 (5901.5–11,436.25) [104]	9480 (6335.5–13,751) [319]	0.044	1.00005 (1.00001–1.0001)
Lymphocytes in units by mm^3^	810.5 (483.5–1120.5) [104]	687 (385–1150) [319]	0.256	1.00 (1.00–1.00)
Monocytes in units by mm^3^	380 (271–633) [102]	426 (282–750) [317]	0.215	1.00 (1.00–1.00)
Neutrophils in units by mm^3^	9400 (6499–12,578.25) [104]	10250 (7138–15,178) [319]	0.057	1.00 (1.00–1.00)
Platelet in units/1000 by mm^3^	234 (190.5–299) [103]	215 (167.25–292) [316]	0.040	0.998 (0.996–1.00)
Neutrophils Lymphocytes Ratio	11.63 (7.86–17.65) [104]	14.67 (8.96–23.5) [319]	0.017	0.999 (0.996–1.002)
Platelet Lymphocytes Ratio	299.43 (209.61–477.12) [104]	308.97 (191.26–483.73) [318]	0.798	1.00 (1.00–1.00)
Creatinine in mg/dL	0.81 (0.61–1.09) [104]	1.22 (0.85–2.24) [318]	<0.001	1.52 (1.21–1.91)
Albumin in mg/dl	3.23 (2.85–3.56) [84]	3.13 (2.65–3.44) [265]	0.060	1.01 (0.97–1.05)
Glutamic-oxaloacetic transaminase in U/L	50.1 (37.98–73.18) [100]	52.9 (33.8–85.6) [283]	0.845	1.00 (1.00–1.01)
Glutamic-pyruvic transaminase in U/L	45.05 (28.45–74.7) [100]	37.15 (22.4–59.68) [282]	0.061	1.00 (1.00–1.00)
Lactic dehydrogenase in U/L	562 (432.5–670) [87]	615 (453–856) [233]	0.016	1.00 (1.00–1.01)
C-reactive protein in mg/dL	12.64 (7.41–19.1) [102]	13.4 (6.95–21.69) [300]	0.499	1.01 (0.99–1.04)
D-dimer in ng/mL	1135 (628.5–4063) [91]	2381 (826.2–6545) [263]	0.009	1.0003 (0.999–1.0001)
Interleukin 6 in pg/mL	48.7 (26.57–142.68) [76]	89.37 (40.86–178.4) [219]	0.024	0.9999 (0.9995–1.0003)
Prototombin activation time in %	100.00 (96.5–100) [98]	96.00 (75–100) [305]	<0.001	0.97 (0.96–0.99)
International Normalized Ratio	1.00 (1.00–1.02) [98]	1.01 (1.00–1.12) [303]	<0.001	5.32 (1.07–26.51)
Neutrophils Lymphocytes derivate Ratio	7.33 (5.25–10.11) [104]	7.33 (5.25–11.5) [319]	0.180	1.04 (1.00–1.08)
Monocytes Lymphocytes Ratio	0.60 (0.33–0.8) [102]	0.67 (0.33–1.18) [317]	0.049	1.48 (1.07–2.06)
Neutrophils Platelet Ratio	38.80 (28.52–51.39) [103]	47.35 (33.09–67.27) [316]	0.001	1.02 (1.01–1.03)
Systemic immune-inflammation index	2.86 (1.63–4.54) [104]	3.31 (1.70–5.39) [319]	0.187	0.99 (0.98–1.01)
Length of stay at the Hospital in days	31.5 (22.75–48.50) [104]	15.50 (7.00–27) [320]	<0.001	

**Table 4:** Logistic regression models and odds ratios related to death in patients with COVID-19 in an adult intensive care unit, evaluated in different scenarios (models for all patients or only for patients on invasive mechanical ventilation).

Model Applied to All Patients				
	Full Multiple Model		Reduced Multiple Model	
Traits Included	*p*-Value	Adjusted Odds Ratio (95% Confidence Interval)	*p*-Value	Adjusted Odds Ratio (95% Confidence Interval)
Invasive mechanical ventilation use	<0.001	351.70 (95.94–1289.22)	<0.001	306.74 (87.47–1075.71)
Age in years	<0.001	1.05 (1.03–1.07)	<0.001	1.04 (1.03–1.06)
Simplified Acute Physiology Score 3 score	0.001	1.03 (1.01–1.05)	0.001	1.03 (1.01–1.04)
Length of stay at the ICU in days	<0.001	0.96 (0.95–0.98)	<0.001	0.96 (0.95–0.98)
Asthma presence	0.299	6.13 (0.20–187.18)		
Chronic kidney disease presence	0.331	1.80 (0.55–5.93)		
Diabetes mellitus presence	0.288	1.44 (0.74–2.80)		
COVID-19 vaccine previous hospital admission	0.491	1.29 (0.62–2.68)		
Obesity presence	0.415	1.26 (0.72–2.22)		
Smoking habit presence	0.728	1.16 (0.51–2.61)		
Time in days from symptom to ICU admission	0.701	1.01 (0.96–1.06)		
Etilism habit presence	0.995	1.00 (0.27–3.61)		
Cardiovascular disease presence	0.791	0.87 (0.32–2.36)		
Chronic obstructive pulmonary disease presence	0.616	0.75 (0.24–2.33)		
Admitted from another service	0.118	0.64 (0.36–1.12)		
Systemic arterial hypertension presence	0.095	0.59 (0.32–1.10)		
Model applied to patients in invasive mechanical ventilation				
	Full Multiple model		Reduced multiple model	
Traits included	*p*-value	Adjusted Odds Ratio (95% Confidence Interval)	*p*-value	Ajusted Odds Ratio (95% Confidence Interval)
Use of vasopressin	<0.001	7.49 (3.29–17.05)	<0.001	7.87 (3.54–17.46)
Renal replacement therapy	<0.001	5.19 (2.23–12.09)	<0.001	5.42 (2.55–11.51)
Red cell distribution width >15	0.011	3.52 (1.34–9.26)	0.003	3.84 (1.60–9.21)
Use of hydrocortisone	0.030	2.57 (1.10–6.03)	0.038	2.33 (1.05–5.16)
Age in years	0.041	1.03 (1.00–1.05)	0.006	1.03 (1.01–1.05)
Days of invasive mechanical ventilation use	<0.001	0.94 (0.92–0.96)	<0.001	0.95 (0.93–0.97)
Admitted from another service	0.026	0.43 (0.21–0.90)	0.020	0.43 (0.21–0.87)
Female sex	0.035	0.47 (0.23–0.95)	0.010	0.42 (0.22–0.82)
Use of noradrenaline	0.060	15.67 (0.90–274.17)		
Neutrophil to platelet ratio abnormal	0.088	2.18 (0.89–5.32)		
Diabetes mellitus presence	0.492	1.54 (0.45–5.22)		
Haematocrit abnormal	0.478	1.30 (0.63–2.67)		
Smoking habit presence	0.733	1.19 (0.44–3.18)		
Time from symptom to ICU admission	0.510	1.02 (0.96–1.09)		
Simplified Acute Physiology Score 3 score	0.527	1.01 (0.98–1.03)		
Total number of comorbidities	0.712	0.91 (0.55–1.50)		
Chronic kidney disease presence	0.862	0.84 (0.13–5.68)		
Chronic obstructive pulmonary disease presence	0.675	0.72 (0.16–3.29)		
Use of Fentanyl	0.050	0.13 (0.02–1.00)		

## Data Availability

The data underlying this article were provided by the Federal University of Uberlândia. Data can be shared according to regular procedures of the university ethics committee.
